# Cushing’s disease due to a pituitary adenoma as a component of collision tumor: A case report and review of the literature

**DOI:** 10.1186/s13256-020-02382-0

**Published:** 2020-05-19

**Authors:** Emre Gezer, Zeynep Cantürk, Alev Selek, Berrin Çetinarslan, İlhan Tarkun, Mehmet Sözen, Umay Kiraz, Yeşim Saliha Gürbüz, Savaş Ceylan, Burak Çabuk

**Affiliations:** 1 Department of Endocrinology and Metabolism, Kocaeli University, Faculty of Medicine, 41380 Kocaeli, Turkey; 2 Department of Endocrinology and Metabolism, Anadolu Medical Center, Kocaeli, Turkey; 3 Department of Pathology, Kocaeli University, Faculty of Medicine, Kocaeli, Turkey; 4 Department of Neurosurgery, Kocaeli University, Faculty of Medicine, Kocaeli, Turkey

**Keywords:** Cushing’s disease, Adenoma, Meningioma, Collision tumor

## Abstract

**Background:**

The coexistence of two morphologically different tumors attached to each other creates a very rare type of tumor called a collision tumor. Collision tumors containing pituitary adenoma–sellar meningioma have only been described in four cases to date; we discuss a fifth case harboring a collision tumor comprising a pituitary corticotroph adenoma and a sellar meningioma in the same anatomic position.

**Case presentation:**

A 34-year-old Caucasian woman presented with menstrual irregularity, severe weakness of the proximal muscles, and 10–15 kg weight gain within a year. Basal plasma cortisol and adrenocorticotrophic hormone levels were 17.7 mg/dL and 58 pg/mL, respectively. Her diurnal cortisol rhythm was impaired (plasma cortisol at 23:00, 18.2 mg/dL) and after a 48-hour, 2-mg dexamethasone suppression test, plasma cortisol level was 13.6 mg/dL. The results were consistent with a diagnosis of Cushing’s syndrome. We then performed a nocturnal 8-mg dexamethasone suppression test and the suppression of cortisol was not greater than 50% (21.4 to 19.3). A pituitary magnetic resonance imaging revealed a tuberculum sellae meningioma arising from within the sellar region. An operation was chosen in order to examine whether the tumor was an adrenocorticotrophic hormone/corticotropin-releasing hormone-secreting lesion or if there were any microadenomas that could be observed during the operation. Via an extended endoscopic endonasal approach the meningioma was resected successfully. Unexpectedly, our patient complained of nausea and vomiting postoperatively. Plasma cortisol was 2.6 mg/dL and orally administered hydrocortisone treatment was initiated immediately. Histopathological examination revealed that the tumor generally consisted of a pituitary corticotroph adenoma infiltrated by meningioma. Our patient maintained hydrocortisone treatment for 11 months. At the latest visit, she had lost 12 kg, and her hypertension, menstrual irregularity, and weakness of the proximal muscles had disappeared. Her mental and physical wellbeing were restored.

**Conclusions:**

To the best of our knowledge, this is the first report of Cushing’s disease due to a pituitary corticotroph adenoma adjacent to a meningioma. Even if a high-dose dexamethasone suppression test fails to suppress basal cortisol level, the importance of considering a suprasellar/sellar meningioma a possible component of a collision tumor presenting as adrenocorticotrophic hormone-dependent Cushing’s syndrome is highlighted here.

## Background

Meningiomas are generally slow-growing and benign tumors of adults and account for approximately 25% of all intracranial tumors [1]. They typically occur in brain parenchyma; however, they can also be in sella turcica, representing approximately 1% of all sellar masses [2]. Suprasellar/sellar meningiomas show a female preponderance and their clinical presentation can range from incidental finding to symptomatic, such as hypopituitarism, visual field disturbances, hyperprolactinemia due to stalk effect, or a combination of these findings [3, 4]. The coexistence of two morphologically different tumors attached to each other is called collision tumor and collision sellar tumors are very rare. In a series of 548 transsphenoidally resected pituitary adenomas, collision tumors accounted for 1.46% [5]. Collision tumors containing pituitary adenoma–sellar meningioma have been described in only four cases to date [6–8]. As the fifth case, we present a case harboring a collision tumor comprising a pituitary corticotroph adenoma and a sellar meningioma in the same anatomic position.

## Case presentation

A 34-year-old Caucasian woman presented with menstrual irregularity, severe weakness of the proximal muscles in both upper and lower extremities, and 10–15 kg weight gain within a year. She had a history of hypertension which was under control by indapamide sustained-release 1.5 mg/day treatment for the past 2 years. She was single and had been working at a hospital’s management services. She had no history of smoking tobacco or alcohol consumption. She also denied exposure to any chemicals or radiation. She reported no family history of any endocrinopathies. Her mother had history of essential hypertension for the past 15 years. On examination, she was awake and oriented in time and space with no cranial nerve deficit. The proximal muscle strength of both upper and lower extremities was 3–4/5; the distal muscle strength of both upper and lower extremities was 5/5. No pathological finding was reported during an examination of upper and lower limbs’ reflexes. Her pulse was 80 beats/minute and blood pressure was 140/90 mmHg; however, she had truncal obesity even if body mass index was 28 kg/m^2^, moon face with plethora, thin skin, and hirsutism on her chest and abdomen. She had no striae or ecchymosis on any limb. Her chest and cardiovascular examinations revealed no abnormality. Her bone mineral density had decreased to − 3.0 SD of Z-score.

**Fig. 1:**
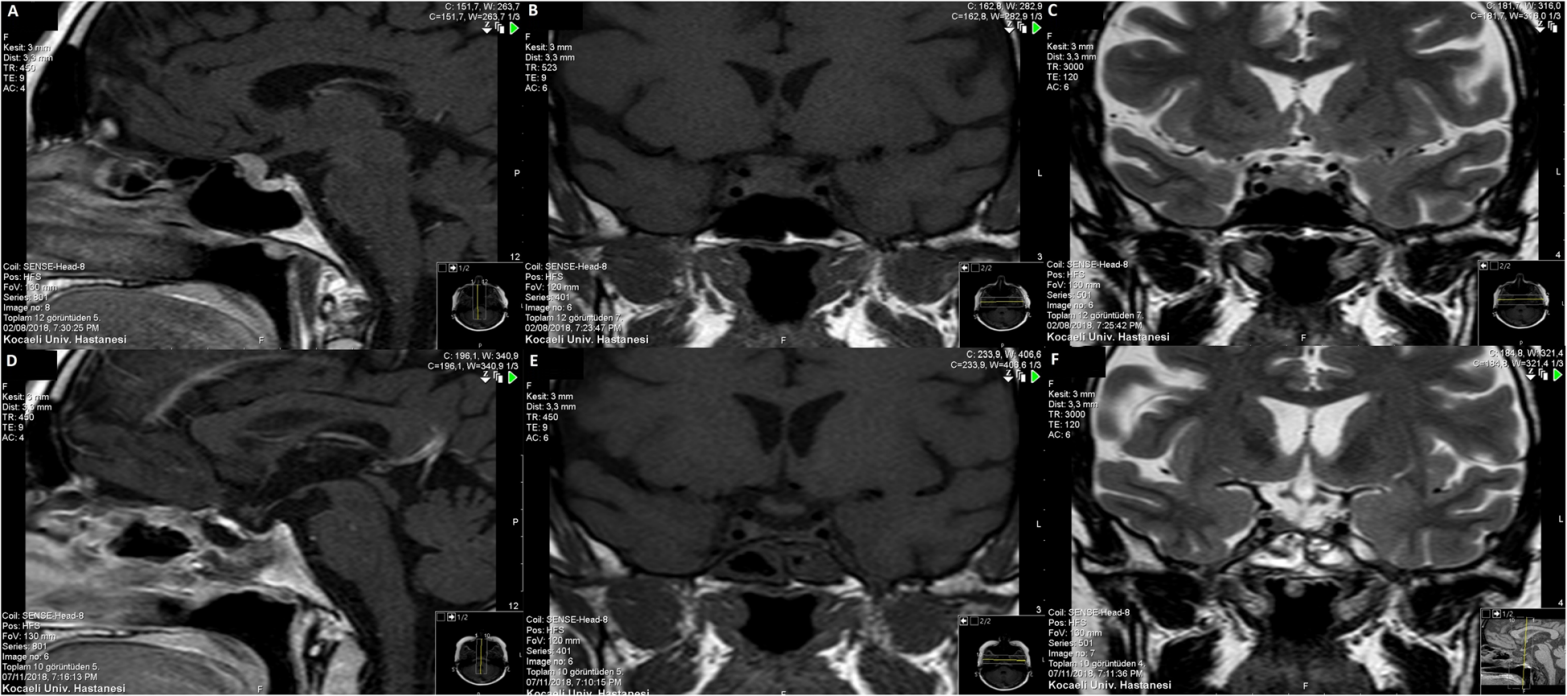
Preoperative and postoperative gadolinium-enhanced magnetic resonance images of the sellar region. **a**–**c** Before transsphenoidal surgery, T1-weighted with contrast sagittal, T1-weighted without contrast coronal, T2-weighted without contrast coronal views, respectively, suprasellar/sellar lesion, in close relation to optic chiasm. **d**–**f** After transsphenoidal surgery, T1-weighted with contrast sagittal, T1-weighted without contrast coronal, T2-weighted without contrast coronal views, respectively, tumor resection

Initial laboratory tests revealed serum thyroid-stimulating hormone level of 2.94 μIU/mL (range 0.38–5.33), free T4 level of 0.68 ng/dL (range 0.6–1.2), and free T3 level of 4.04 pg/mL (range 2.6–4.4). Follicle-stimulating hormone, luteinizing hormone, and estradiol levels were within normal range. Basal plasma cortisol and adrenocorticotrophic hormone (ACTH) levels were 17.7 mg/dL and 58 pg/mL, respectively. Her 24-hour urine free cortisol was 874 nmol/24-hour (range < 403). Her diurnal cortisol rhythm was impaired (plasma cortisol at 23:00, 18.2 mg/dL) and after a 48-hour, 2-mg dexamethasone suppression test, plasma cortisol level was 13.6 mg/dL. The results were consistent with a diagnosis of Cushing’s syndrome. We then performed a nocturnal 8-mg dexamethasone suppression test and the suppression of cortisol was not greater than 50% (21.4 to 19.3). The following night, an 8-mg dexamethasone suppression test was repeated, and the suppression of cortisol was still less than 50% (21.4 to 12.5). A pituitary magnetic resonance imaging (MRI) revealed a solid mass with well-defined margin in the anterior part of the gland, which measured approximately 12 × 7.5 mm maximum diameter, and suggested a tuberculum sellae meningioma arising from within the sellar region (Fig. 1). A visual field test was performed due to the close relationship between the lesion and optic chiasm, but the results were normal. At admission, her potassium level was 2.9 mmol/L and intravenously administered potassium supplementation (60 mEq/day) was required daily to keep potassium levels normal. Her indapamide treatment was replaced by ramipril 5 mg once a day.

**Fig. 2:**
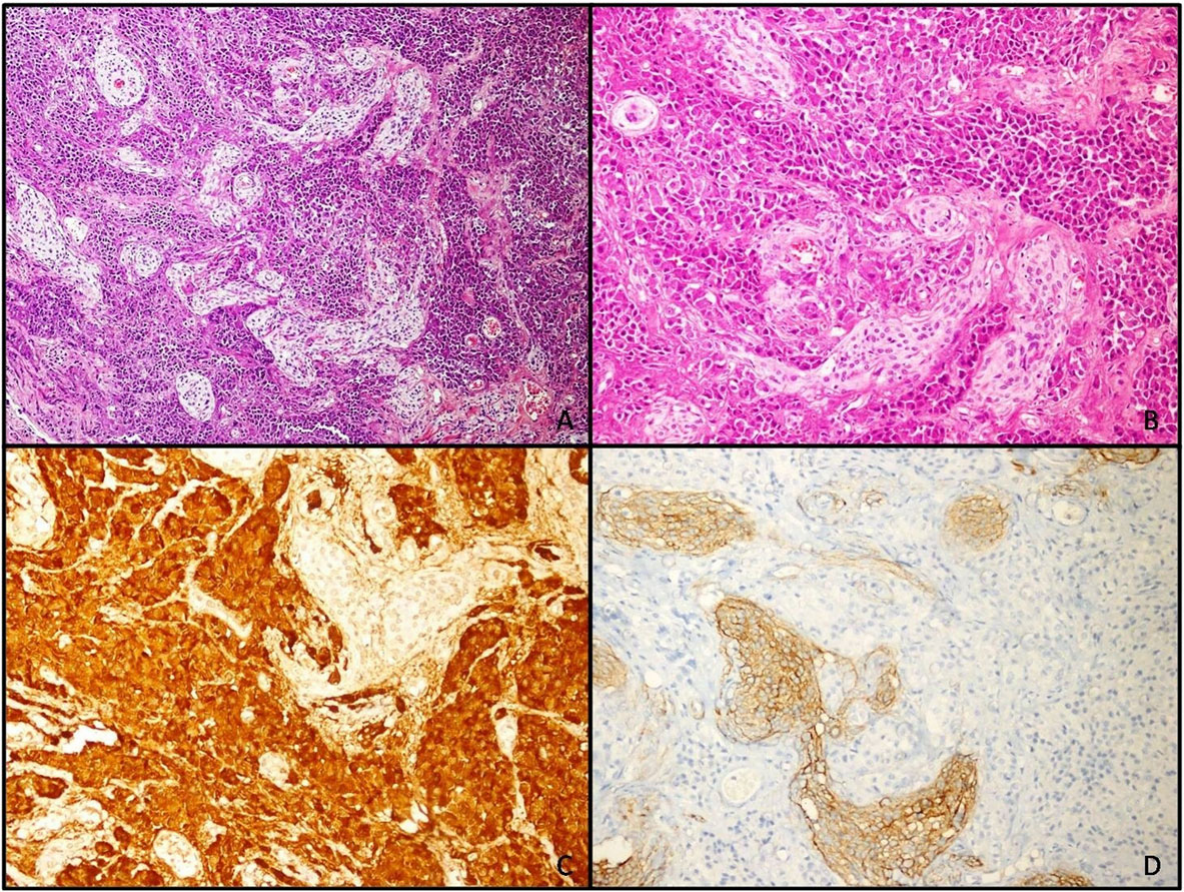
Histopathological and immunohistochemical evaluation. Hematoxylin and eosin of pituitary adenoma and meningioma, × 100 (**a**), × 200 (**b**), adrenocorticotrophic hormone staining in pituitary adenoma, × 200 (**c**), and epithelial membrane antigen staining in meningioma, × 200 (**d**)

High-resolution computed tomography (CT) of her lungs, ^18^F-fluorodeoxyglucose (^18^F-FDG) positron emission tomography (PET)/CT, ^68^Ga-DOTATATE PET/CT, and upper endoscopy/colonoscopy were performed to rule out any ectopic ACTH-secreting tumor and there was no pathological finding reported. A corticotropin-releasing hormone (CRH) test and inferior petrosal sinus sampling (IPSS) were not performed despite the absence of any displayed pituitary tumor, because it was decided to operate on the meningioma because of its size and localization. An operation was also chosen in order to examine whether the tumor was an ACTH/CRH-secreting lesion or if there were any microadenomas that could be observed during the operation. In addition, CRH ampul is not available in our country and it may often be challenging to provide it. Finally, via an extended endoscopic endonasal approach the meningioma was resected successfully. Unexpectedly, our patient complained of nausea and vomiting postoperatively. Her plasma cortisol and ACTH levels were 2.6 mg/dL and 39 pg/mL, respectively and oral hydrocortisone treatment (20 mg/day) was initiated immediately. A histopathological examination revealed an unexpected appearance. The tumor generally consisted of a pituitary adenoma including uniform cell proliferation which was infiltrated by meningioma in small groups and storiform patterns. On immunohistochemical examination, the pituitary adenoma was strongly and diffusely stained with ACTH. Growth hormone (GH) and p53 staining was not detected; Ki67 index was approximately 2%. The meningioma cells were stained strongly with epithelial membrane antigen (EMA) and progesterone receptor (PR). This staining pattern was in the form of a mirror. A biopsy was reported as a collision tumor composed of pituitary corticotroph adenoma according to the 2017 World Health Organization classification of pituitary adenoma [9] and meningioma (Fig. 2). Our patient maintained hydrocortisone treatment (20 mg/day) for 8 months and treatment was tapered to a stop over the final 3 months. At the latest visit, she had lost 12 kg of weight, and her hypertension, menstrual irregularity, and weakness of the proximal muscles had disappeared. A laboratory evaluation had no pathological finding including plasma ACTH level which was 30 pg/mL. Her mental and physical wellbeing were restored.

## Discussion

As noted above, the etiology of Cushing’s syndrome in our patient turned out to be a pituitary corticotroph adenoma which was a component of a collision tumor comprising meningioma as well. It was demonstrated by both the immunohistochemical examination and the adrenal insufficiency developed after surgical resection of the meningioma. To date, this is the first case report describing a patient with Cushing’s disease due to a pituitary corticotroph adenoma located in the same anatomic position as a suprasellar/sellar meningioma, which were the components of a collision tumor.

Due to their similar imaging characteristics, suprasellar/sellar meningiomas can mimic pituitary adenomas radiologically [10]. Kwancharoen *et al.* examined 57 cases of suprasellar/sellar meningiomas and 11% of them were diagnosed incorrectly as pituitary adenoma [11] or vice versa. The MRI findings of our patient were reported as tuberculum sellae meningioma arising from within the sella; on the other hand, the histopathology report described the lesion as meningioma and pituitary corticotroph adenoma located in the same anatomic position. She had no history of receiving any radiotherapy for a pituitary mass.

Because the actual definition of a collision tumor is a lesion of two separate tumor types along a shared border, as described in our patient, the diagnosis of a collision sellar lesion can only be determined by histological examination; therefore, preoperative diagnosis is quite difficult. Most case reports include a pituitary adenoma coexisting with either neoplastic, adenomatous, congenital, vascular, or inflammatory sellar lesions such as: another adenoma [5, 6, 12, 13]; craniopharyngioma [14–18]; schwannoma [5]; hypophysitis [19, 20]; arachnoid, colloid, and epidermoid cysts [21–23]; gangliocytoma [5, 24–26]; Rathke’s cleft cyst [5, 14, 27]; neurosarcoidosis [5, 28]; plasmacytoma [29]; chondroma [30]; lymphoma [31]; lung cancer metastasis [32]; and meningioma [6–8].

In the literature, four cases of collision sellar tumors with pituitary adenoma–sellar meningioma were previously described, as listed in Table 1. The first one was reported in 1984 by Banik *et al.* [6]; the patient was diagnosed as having multiple endocrine neoplasia type 1 demonstrating collision tumors of both pituitary and adrenal glands. After she died of aspiration bronchopneumonia, an autopsy revealed an asymptomatic pituitary tumor composed of three components: meningioma, chromophobe, and acidophil adenomas. Second, Karsy *et al.* reported a non-secreting pituitary adenoma that coexisted with sellar meningioma in 2015 [7]. Last, two patients with acromegaly were described by Zhao *et al*. and their histopathological reports displayed collision tumors with GH-secreting pituitary adenomas and sella meningiomas [8]. On the other hand, plenty of case reports described various coexistent pituitary adenoma and isolated suprasellar meningioma [33–38]. At this point, the difference between “coexistent/concomitant” and “collision” should be underlined. A “collision” tumor has two different lesions in the same anatomic position, whereas “coexistent/concomitant” tumors are two different lesions at two separate locations.

**Table 1:** Four cases of collision sellar tumors with pituitary adenoma–sellar meningioma

Authors and reference number	Age	Sex	Clinical presentation	Histology
Banik *et al*. [6]	56	F	Persistent heartburn and vomiting	Non-secreting PA + GH-secreting PA + meningioma
Karsy *et al*. [7]	70	F	Altered mental status, mutism, and incontinence	Non-secreting PA + meningioma
Zhao *et al*. [8]	58	F	Acromegaly	GH-secreting PA + meningioma
Zhao *et al*. [8]	58	F	Acromegaly	GH-secreting PA + meningioma

*F* female, *GH* growth hormone, *PA* pituitary adenoma

Preoperatively, the tuberculum sellae meningioma described in MRI findings of our patient was considered a possible etiology of Cushing’s disease. To date, only two cases with meningioma-induced Cushing’s syndrome have been reported (in 2003 and 2014) [39, 40] and, in contrast to our case, they both were with parietal meningiomas secreting CRH.

Unlike collision tumors, the coexistence of pituitary adenoma and intracranial meningioma outside the sella is not uncommon [33]. Predisposing conditions such as radiation, trauma, genetic factors, and chronic inflammation should be considered. Besides these factors, the *in vivo* mechanism of a collision tumor comprising a pituitary adenoma and meningioma is not fully understood. One suggested mechanism is that GH secretion induces meningioma formation in patients with GH-secreting pituitary adenoma [41, 42]. Other possible etiologies beyond stimulation by GH can be paracrine growth effects of other pituitary hormones, due to hormone receptor immunoreactivity found within the meningiomatous component of the collision tumor or the existence of a common progenitor cell of origin as described in other types of collision tumors [31, 43].

## Conclusions

To the best of our knowledge, our case report appears to be the first one with Cushing’s disease due to a pituitary corticotroph adenoma located in the same anatomic position as a suprasellar/sellar meningioma, which are the components of a collision tumor. Even if a high-dose dexamethasone suppression test fails to suppress basal cortisol level, the importance of considering a suprasellar/sellar meningioma as a possible component of a collision tumor presenting as ACTH-dependent Cushing’s syndrome is highlighted here. There is still no proven pathogenetic mechanism explaining the relationship between a pituitary adenoma and a sellar meningioma located in the same lesion. Additional examinations such as chromosomal analysis with electron microscopy and immunohistochemical stainings are needed to elucidate the responsible mechanisms. Transsphenoidal surgery remains the gold standard treatment for any type of suprasellar/sellar lesion suspected from imaging findings; however, it should be undertaken by only experienced neurosurgeons due to high risk of intraoperative bleeding because of their invasiveness into adjacent structures and distinct vascular supply.

## Data Availability

All of the data and materials will be available upon request to the corresponding author.

## Abbreviations

^18^F-FDG: ^18^F-fluorodeoxyglucose
ACTH: Adrenocorticotrophic hormone
CRH: Corticotropin-releasing hormone
CT: Computed tomography
EMA: Epithelial membrane antigen
GH: Growth hormone
IPSS: Inferior petrosal sinus sampling
MRI: Magnetic resonance imaging
PET: Positron emission tomography
PR: Progesterone receptor
